# Effect of Peer Attachment on Legal Emotion Among Junior Middle School Students: The Mediation Role of Self-Esteem

**DOI:** 10.3389/fpsyg.2022.923604

**Published:** 2022-07-01

**Authors:** Shuhui Xu, Lu Fan, Chunjing Su

**Affiliations:** ^1^Department of Psychology, School of Education, Wenzhou University, Wenzhou, China; ^2^School of Educational Science, Ludong University, Yantai, China

**Keywords:** legal consciousness, legal emotion, development, self-esteem, peer attachment, prosocial tendencies, interpersonal trust

## Abstract

Legal consciousness is the individual consciousness which reflects legal phenomena. Well-developed legal consciousness plays a crucial role in informing citizens of his legal right and ability to exercise these rights, therefore forms certain connections of individuals and society, and its development is a key part of individual’s socialization process. Here, we investigated the emotion aspect of legal consciousness (henceforth legal emotion) and tried to identify several factors affecting the development of legal emotion and several factors affected by legal emotion. A large sample of Chinese junior middle school students (*N* = 967) completed a battery of self-reported questionnaires regarding legal emotion, peer attachment, self-esteem, prosocial tendencies, and interpersonal trust. The results indicated that for early adolescents, peer attachment predicts both positive and negative legal emotion. Importantly, peer attachment affects legal emotion partially through self-esteem. We also showed that negative legal emotion acted as a mediator on the relationship between interpersonal trust and prosocial tendencies. The results provided important insights into the role of legal emotion, the emotion aspect of legal consciousness, in the social interactions and its potential psychological mechanism.

## Introduction

Legal consciousness is the individual consciousness that reflects legal phenomena (Chua and Engel, 2019). It is a complex socio-psychological phenomenon, which includes philosophical, legal, socio-political, and moral aspects. Well-developed legal consciousness plays a crucial role in informing citizens of their legal rights and ability to exercise these rights and is therefore essential for the current renewed society in which drastic social reforms and conceptual changes in the field of law occur.

As a theoretical construct and sociocultural phenomenon, legal consciousness has been a major research interest of various scientific disciplines—jurisprudence, sociology, philosophy, ethics, etc. Psychology does not stand aside. It should be noted that legal consciousness is predominately a subjective, psychological phenomenon: It correlates with the essence of law, a subjective understanding of lawfulness, and the individual law representations in the individual’s consciousness. In this sense, legal consciousness cannot be thoroughly understood without psychological research. Legal psychology is closely related to the legal consciousness: its formation, development, and relationship to other psychological structures are on the list of issues that modern psychology is trying to investigate.

### The Legal Emotion, a Key Component of Legal Consciousness

The psychological research on the structure of legal consciousness has evolved substantially with the first attempts made to bridge the gap between law in the books and law in action (see Silbey, 2005). The Soviet scholar I.E. Farber distinguished between two aspects in the structure of legal consciousness: (1) the cognitive aspect (legal ideology); (2) the emotional-volitional aspect (legal psychology). Legal ideology is understood as a system of legal knowledge whereas legal psychology is regarded as a combination of feelings, beliefs, habits, and motives of legally volitional actions. This was further developed into a three-component-construct: cognitive (legal concepts and knowledge, cognitive assessment of legal phenomena), affective (emotional assessment of legal phenomena), and behavioral (intention to behave in a certain way in legal situations; Molotova et al., 2020). More complex models were also proposed, such as a four-component model of legal consciousness introduced by Shchegortsev (1981), which includes a substantive component (knowledge and ideas of law), evaluative component (assessment of legal phenomena), behavioral component (behavior and activity of people in legal situations) and energy (emotions and feelings of people experienced in a meaningful situation); and a six-component model was proposed by Horák et al. (2021). Together, although there is an ongoing debate on the actual structure of legal consciousness, the majority of the researcher agrees that emotion is a key component of legal consciousness. In the present manuscript, we aim to focus on the emotional aspect of legal consciousness (henceforth “legal emotion”).

Emotion was initially introduced as a conceptual component of the legal socialization process (Hogan and Mills, 1976): e.g., adolescents feel guilty or good when they violate or follow the rules because they feel social disapproval or approval from their peers. In another word, emotion was largely relegated to the role of internal drivers of cognitive processes such as rational and deliberative processes. In this sense, the traditional psychological viewpoints (from Jean Piaget to Lawrence Kohlberg, as well as some other psychologists such as James Rest and Carol Gilligan, for a review, see Fleming, 2005) is that the development of moral feeling is fundamentally the amount of cognitive development. Consequently, emotions were overlooked in their own presence.

However, emotion is a key human component in the process of reasoning, forming attitudes, and making decisions. It has been studied within the context of many relevant areas, such as socio-legal issues (e.g., Bandes and Blumenthal, 2012) and delinquency (e.g., Spruit et al., 2016). Importantly, inconsistent with the above-mentioned traditional psychological viewpoints, the modern dual-process theory of morality suggested that emotional and deliberate reasoning are not only qualitatively distinctive but also compete in making moral decisions. In this sense, the unique contribution of emotion to the legal socialization of children deserved further investigation.

The social-learning theory-makers, i.e., Bandura (1977) believes that moralities are compatible with social norms. These theories emphasized the distinction between the children’s moral competencies and their real behaviors. The competencies included his/her awareness of moral laws and rules and his/her abilities in creating moral behaviors. Bandura posits that the basis of morality and moral behavior is in social learning, imitation, and modeling after others. Moral behaviors, such as his/her reasoning’s about social rules and laws, are acquired mainly through strengthening, punishment, and imitation during social interactions. The social-learning theories postulate that legal socialization depends not only on the legal authorities but also on non-legal authorities, such as parents and teachers. However, previous investigations have largely neglected to acknowledge the effect of non-legal authorities such as peers. The purpose of the current paper is to establish an initial theoretical model of peer interaction and the development of legal emotion.

The concept of emotion encompasses a complex array of human internal experiences. It has different definitions across different disciplines and among researchers (Izard, 2010). In the present study, we define legal emotion as the legal consciousness that reflects the overall emotional assessment of law as a sociocultural phenomenon. That is, we define legal emotion as explicit emotional assessments of law rather than the subjective internal emotional experience of law phenomena. The benefit of this definition will be that it allows us to use self-reported questionnaires to measure the legal emotion of an individual.

### The Peer Attachment and the Legal Emotion in Early Adolescents

Legal consciousness keeps forming throughout the development, enriched *via* the more complex activities and communications. Nonetheless, its development in early adolescence might be of particular interest. What is especially important is that at this stage of development, children turn their attachment relationship, especially the emotional bond of attachment, from caregivers to peers (Hazan and Shaver, 1994; Paterson et al., 1994). It is well documented that social institutions (schools, entertainment organizations, teams of colleagues, etc.) play an essential part in the legal socialization and moral formation of a child, and being involved in more complex activities and communication enriches the legal aspects of their consciousness.

In this sense, the interaction with peers might have an important effect on the formation of their legal consciousness. Moreover, since an important aspect of caregiver to peer transaction is the emotional bond of attachment shifting from caregivers to peers, it would be of particular interest to explore how the quality of peer attachment affects the formation of the emotional aspect of legal consciousness, i.e., the legal emotion. In the present study, we aim to investigate this topic.

We further aim to explore the underlying mechanism of this relationship, such as if it is mediated through self-esteem. It is established that there is a strong relationship between security in attachment and self-esteem, both for parent attachment and peer attachment (Gorrese and Ruggieri, 2013). At the same time, there is a strong relationship between rational self-esteem and general self-consciousness (Kim et al., 2019) and emotional response (Laible et al., 2004; Ju and Lee, 2018) in adolescents. Therefore, self-esteem might act as a potential mediation factor between peer attachment and the emotional aspect of legal consciousness. Previous studies found that self-esteem act as a mediator between peer attachment and many self-related psychological variables (e.g., distress, Imran and Jackson, 2022; temperament, Remondi et al., 2022; self-concept, Wu, 2009; relationship satisfaction, Sisi et al., 2021). Understanding the underlying mechanism between peer attachment and legal emotion has the potential to aid in the development of educational and intervention strategies for the forming of legal consciousness among young adolescents.

### The Role of Legal Emotion in Prosocial Behavior

Interpersonal trust is a measure of peer relationships. When peers are trustworthy, relationships are closer and prosocial behavior is more likely. In contrast, when peer trust is low, relationships are more distant, resulting in less significant cooperation tendencies (Wang and Chen, 2011). Accordingly, it can be posited that interpersonal trust positively affects prosocial behavior (Acedo-Carmona and Gomila, 2019).

We hypothesize that, for the peers, the legal emotion, especially the negative legal emotion, might play a potential role between the interpersonal trust and prosocial behavior. Being wronged by a member of a group to which we belong occurs frequently during social interactions and under what circumstance might we view law (or regulations) as relevant in such unfair dispute? In such a situation the emotions are particularly salient. Previous research has highlighted the role of emotions in the development or non-development of such disputes, and it shows that the emotion can largely determine how and when the law becomes relevant in such a scenario (Wang, 2019). Obviously, in such a scenario, different levels of interpersonal trust might regulate the evoked emotions: for example, people are more easily angry when the offender is a stranger. In this case, the emotion might act as a mediator between interpersonal trust and prosocial behavior. Here, we aim to test this hypothesis empirically. Given that in the above described scenario, only negative emotion will be evoked, we only test the negative legal emotion in the present study.

### The Present Study

The current study aimed to examine two questions. First, what factors affect the junior middle students’ legal emotions? More specifically, we aim to examine the effects of peer attachment and self-esteem on legal emotions and test whether self-esteem plays a mediating role in the process by which peer attachment influences legal emotions. Second, how will legal emotion interact with our social interactions? More specifically, we aim to test whether legal emotion plays a mediating role in the process by which interpersonal trust influences prosocial tendencies. We present our results below.

## Materials and Methods

### Participants

One thousand hundred adolescents, from 3 typical public junior middle schools located in the Wenzhou City, southeastern regions of China (500,300,300 questionnaires were distributed in each middle school, respectively) took part in the survey. In total, 967 valid questionnaires were obtained (422,256,289 samples of each school, respectively). The sample size was determined following Ghiselli et al.’s sampling criteria (1981) that the study sample size should be 10 times the total number of questions (Ghiselli et al., 1981). Their sociodemographic characteristics are summarized in Table 1 below.

**Table 1:** Sociodemographic characteristics of the sample (*N* = 967).

	Groups	*N*	%	M(SD)
Age	11–14 years	967		12.9(1.52)
Gender	Male	540	55.8%	
	Female	427	44.2%	
Academic year	First year (grade 8)	561	58.0%	
	Second year (grade 9)	406	42.0%	
Only-child	YES	493	51.0%	
	NO	474	49.0%	
Self-reported Parents‘education level	Both Bachelor’s or above	177	18.3%	
	Only one Bachelor’s or above	115	11.9%	
	Both high school or equivalent	263	27.2%	
	Only one high school	215	22.2%	
	Below high school (or unknown)	197	20.4%	
Living Area	Rural	545	56.4%	
	Urban	422	43.6%	

### Measurements

#### Measurement of Legal Emotion

The middle school student’s legal emotion questionnaire is a self-report questionnaire that was constructed previously by the author (Xu, 2020) to assess and estimate the attitudinal or emotional relations of an individual toward law. It has 43 items with two dimensions: the positive emotion (20 items, including interesting, anticipating, and confidence) and negative emotion (23 items, including contempt, disgust, and disappointment). Items were rated using a 5-point Likert scale, ranging from 1 = strongly disagree, to 5 = strongly agree.

The scale has shown good internal consistency (Cronbach alpha scores: 0.957 for the positive emotion sub-scale, 0.934 for the negative emotion sub-scale). In our sample, the internal consistency of the peer version was *α* = 0.902 (positive emotion sub-scale = 0.90; negative emotion sub-scale = 0.94). In line with these results, in our sample the Cronbach’s alphas were excellent.

#### Measurement of Peer Relationships

The Revised Inventory of Parent and Peer Attachment (IPPA-R, Armsden and Greenberg, 1987) is a self-report questionnaire that was constructed for people between 12 and 19 years to assess and estimate the quality of attachment to maternal, paternal, and peers, as reflected in high levels of mutual trust and quality of communication, and low levels of anger and alienation. It uses three scales (25 items each), one for each attachment figure, which have proven to be independent and internally consistently associate positively with the quality of the familiar environment, and negatively with loneliness and hopelessness in adolescents. In the present study, we only use the peer version. The adolescents rate their agreement with each item on a 5-point Likert-type scale (1 = never or almost never true, 5 = always or almost always true). The scale provides a global security attachment score and those on the following three dimensions of the attachment relationship: Trust, Communication, and Disaffection. According to the framework of attachment theory, the score on the disaffection scale is reversed, and thus a low score corresponds to a high level of attachment security and vice versa.

The validation of the Chinese version of the IPPA-R was performed by Zhang et al. (2011). In our sample, the internal consistency of the peer version was *α* = 0.902.

#### Measurement of Self-Esteem

The Rosenberg Self-Esteem Scale RSES (Rosenberg, 1965), a 10-item measure of global self-esteem, was administered. The RSES comprises 10 items and is commonly used as an empirical measure of a person’s overall self-esteem. Items were rated using a 4-point Likert scale, ranging from 1 = strongly disagree, to 4 = strongly agree. The higher the score, the higher the self-esteem.

The RSES has been translated into Chinese and used among Chinese-speaking populations frequently over the last three decades (Wang et al., 2010). The internal consistency in the present sample was *α* = 0.890.

#### Measurement of Interpersonal Trust

The Interpersonal Trust Scale (ITS, Hochreich and Rotter, 1970) has 25 items with two dimensions: trust in relatives or friends and trust in people who have no direct relation. Scores are given on a five-point scale ranging from 1 (Does not describe me at all) to 5 (Describes me well). There are 12 positive items and 13 negative items. The average score for the 26 items was calculated, with a higher score indicating a higher level of prosocial behavior.

The Chinese version of the ITS was applied in this study (revised edition; Wang et al., 1999). The internal consistency in the present sample was *α* = 0.68.

#### Measurement of Prosocial Tendencies

The Prosocial Tendencies Measure (PTM) developed by Carlo and Randall (2002) measuring prosocial behavior tendencies on six subscales: altruistic (5 items), anonymous prosocial behavior (5 items), compliant prosocial behavior (2 items), dire prosocial behavior (3 items), emotional prosocial behavior (4 items), and public prosocial behavior (4 items). The scales were developed for late adolescents. A revised version, consisting of 25 Items, adding one item to altruistic and one item to emotional prosocial behavior was later published (Carlo et al., 2016).

The Chinese version of the PTM (Kou et al., 2007) was used. This instrument has 26 items and uses a 5-point Likert scale ranging from 1 (does not describe me at all) to 5 (describes me very well); higher scores indicate higher levels of prosocial tendencies. In the current study, Cronbach’s α for the scale was 0.941, with each six subscales being 0.815, 0.790, 0.821, 0.813, 0.774, and 0.665.

### Procedure

Data were collected through self-administered paper-and-pencil questionnaires distributed by their teacher during self-study time. The teacher was instructed to clarify that participation in the survey was on a voluntary and anonymous basis. It also was explicitly explained to the students that they had the right to refuse to participate without any penalty.

### Data Analysis

All data analyses were conducted using IBM SPSS 23.0 and the PROCESS macro developed by Hayes (2013). First, common method variance was examined using confirmatory factor analysis. Then we explored the bivariate relationship between main variables through correlation analysis. Finally, we explored the specific relationship between pairs of the variables and examined the mediation effects *via* regression and bootstrap analyses.

## Results

### Common Method Bias

All questionnaires were self-assessment scales that may produce common method bias. Therefore, common method bias was examined using the Harman single-factor test. Exploratory factor analysis was performed without rotation. There are 14 factors with feature values greater than 1 extracted. The explanatory variance of the first factor is 22.4% (which is far less than the critical value of 40%), and the cumulative interpretation total variance was 62.3%. Therefore, the results show that there is no obvious common method bias in this study.

### Description Statistics and Correlation Matrix

The mean, standard deviation, and correlation coefficient of latent variables were statistically analyzed using SPSS 23.0. As shown in Table 2, the mean and standard deviation of each variable were within the acceptable range.

**Table 2:** Descriptive statistics and correlation matrix of main research variables (*N* = 967).

	M ± SD	1	2	3	4	5	6
1. Positive legal emotion	86.09 ± 14.15	1					
2. Negative legal emotion	37.92 ± 18.29	−0.667^***^	1				
3. Peer attachment	87.47 ± 15.84	0.299^***^	−0.273^***^	1			
4. Self-esteem	28.27 ± 6.85	0.321^***^	−0.299^***^	0.431^***^	1		
5. Prosocial tendencies	96.26 ± 16.94	0.298^***^	−0.336^***^	0.317^***^	0.294^***^	1	
6. Interpersonal trust	75.22 ± 9.78	0.359^***^	−0.415^***^	0.311^***^	0.371^***^	0.283^***^	1

*** *p < 0.001.*

Pearson correlations revealed that a significant correlation exists between legal emotion (both positive and negative) and peer attachment, self-esteem, prosocial tendencies, and interpersonal trust. A significant correlation also exists between peer attachment and self-esteem, and between prosocial tendencies and interpersonal trust. Although all the correlations were significant, the strongest correlations were between negative legal emotion and interpersonal trust. The results of descriptive statistics and related analysis preliminarily illustrate the relationship between variables, thus providing a basis for further data analysis.

For demographic variates, Pearson correlation analysis between age and the variables yielded significant, albeit weak, correlations between age and positive legal emotion (ps < 0.01), negative legal emotion (ps < 0.01), and self-esteem (ps < 0.05). Similarly, there’s gender differences in negative legal emotion, self-esteem, peer attachment, prosocial tendencies and interpersonal trust (ps < 0.01). Consequently, age and gender were entered as control variables in further analyses.

### The Mediation Effect of Self-Esteem in the Effect of Peer Attachment on Legal Emotion

Mediation analyses were performed, using the SPSS and the PROCESS macro, to verify the indirect effects of the predictor on the dependent variable. The 95% confidence interval was calculated using the bias-corrected bootstrapping method (*n* = 5,000).

#### Regression Analysis

As shown in Table 3, the peer attachment of middle school students significantly and positively impacted their positive legal emotion (*B* = 0.268, *t* = 5.00, *p* < 0.001). The peer attachment of middle school students also significantly and positively affected self-esteem (*B* = 0.191, *t* = 7.85, *p* < 0.001). After adding the mediating variable self-esteem, peer attachment had a significant positive effect on positive legal emotion (*B* = 0.173, *t* = 2.97, *p* < 0.01) and their self-esteem also significantly and positively influenced positive legal emotion (*B* = 0.497, *t* = 3.67, *p* < 0.001). The B value for the effect of peer attachment on positive legal emotion decreased from 0.268 to 0.173, but was still significant.

**Table 3:** Results of multiple regression analyses.

Variables	Positive legal emotion			Self-esteem			Positive legal emotion		
	*B*	SE	*t*	*B*	SE	*t*	*B*	SE	*t*
constant	71.35	4.209	16.953^***^	22.851	1.910	11.967^***^	59.993	5.145	11.660^***^
Peer attachment	0.268	0.054	5.003^***^	0.191	0.024	7.853^***^	0.173	0.058	2.968^**^
Self-esteem							0.497	0.136	3.667^**^
Gender	−0.568	1.696	−0.335	−2.005	0.769	−2.606^**^	0.428	1.678	0.255
Age	1.628	1.135	1.434	−0.116	0.515	−0.226	1.685	1.108	1.521
*R* ^2^	0.097			0.207			0.143		
*F*	9.018^***^			21.956^***^			10.460^***^		

** *p < 0.01; and*

*** *p < 0.001.*

For negative legal emotion (see Table 4), the peer attachment of middle school students significantly and negatively impacted their negative legal emotions (*B* = −0.313, *t* = −4.46, *p* < 0.001). After adding the mediating variable self-esteem, peer attachment had a significant negative effect on negative legal emotion (*B* = −0.195, *t* = 2.542, *p* < 0.01) and their self-esteem also significantly and negatively influenced negative legal emotion (*B* = −0.621, *t* = −3.49, *p* < 0.001). The value of B for the effect of peer attachment on negative legal emotion decreased but was still significant.

**Table 4:** Results of multiple regression analyses.

Variables	Negative legal emotion			Self-esteem			Negative legal emotion		
	*B*	SE	*t*	*B*	SE	*t*	*B*	SE	*t*
constant	52.582	5.508	9.547^***^	22.851	1.910	11.967^***^	66.761	6.749	9.891^***^
Peer attachment	−0.313	0.070	−4.464^***^	0.191	0.024	7.853^***^	−0.195	0.077	−2.542^*^
Self-esteem							−0.621	0.178	−3.490^***^
Gender	−0.836	2.219	−0.377	−2.005	0.769	−2.606^**^	−2.080	2.201	−0.945
Age	0.407	1.485	0.274	−0.116	0.515	−0.226	0.335	1.453	0.230
*R* ^2^	0.207			0.075			0.118		
*F*	21.956^***^			6.836^***^			8.399^***^		

* *p < 0.1;*

** *p < 0.01; and*

*** *p < 0.001.*

Together, we posit that self-esteem plays a partial mediation role in the effect of peer attachment on both positive and negative legal emotion in middle school students.

#### Validation of the Mediating Role of Self-Esteem

We use PROCESS macro to test the mediation role of self-esteem in the relationship between peer attachment and legal emotion. Two types of legal emotion (positive and negative) were tested separately. In both models, age and gender were included as control variables. Indirect effects are unstandardized coefficients, which are significant when the 95% confident interval does not contain zero.

**Figure 1:**
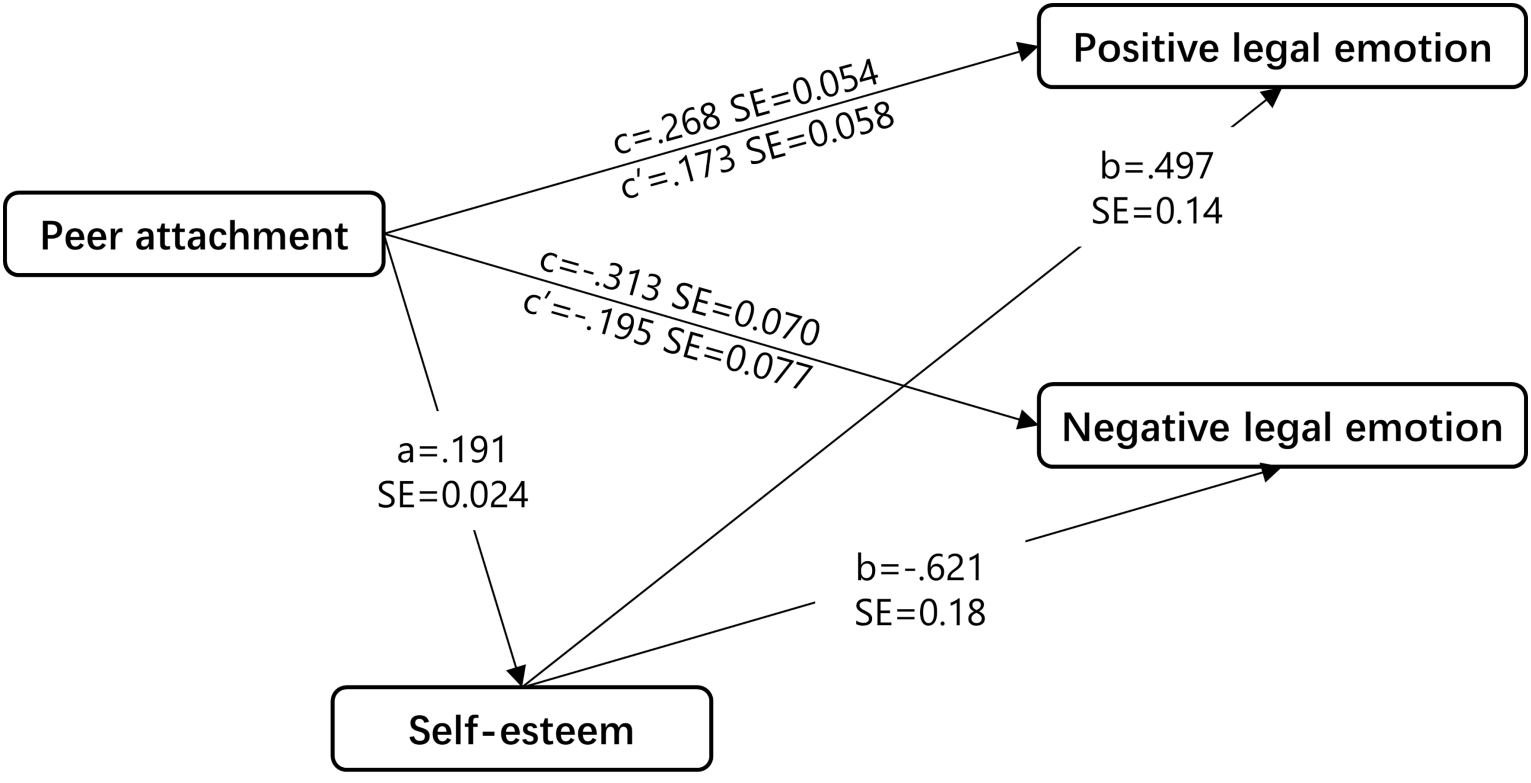
Hypothesized model with self-esteem as the mediator between peer attachment and legal emotion for the entire sample. Unstandardized regression weights are presented. Controlled variables (age and gender) are omitted for clarity. All paths were significant at the *p* < 0.01 level.

Results confirm that that self-esteem partially mediates the relation between peer attachment and positive legal emotion (Figure 1): indirect effect = 0.095, SE = 0.033, 95% CI = [0.023, 0.165]. Therefore, the indirect effect is significantly different from zero at *p* < 0.05. The mediator could account for 35.4% of the effect.

Results show also that that self-esteem partially mediates the relation between peer attachment and negative legal emotion (Figure 1): indirect effect = −0.119, SE = 0.044, 95% CI = [−0.041, −0.215]. The mediator could account for 37.9% of the effect.

Therefore, the results validate that self-esteem plays a partial mediation role in the effect of peer attachment on both positive and negative legal emotion in middle school students.

### The Mediation Effect of Negative Legal Emotion in the Effect of Interpersonal Trust on Prosocial Tendencies in Middle School Students

#### Regression Analysis

As shown in Table 5, the interpersonal trust significantly and positively impacted their prosocial tendencies (*B* = 0.472, *t* = 4.89, *p* < 0.001). The negative legal emotion also significantly and negative affected prosocial tendencies (*B* = −0.509, *t* = −7.61, *p* < 0.001). After adding the mediating variable negative legal emotion, the interpersonal trust had a significant positive effect on prosocial tendencies (*B* = 0.292, *t* = 2.85, *p* < 0.01) and their negative legal emotion also significantly and negatively influenced prosocial tendencies (*B* = −0.353, *t* = −4.26, *p* < 0.001). The B value for the effect of interpersonal trust on prosocial tendencies decreased from 0.472 to 0.292, but was still significant. Therefore, we posit that negative legal emotion plays a partial mediation role in the effect of interpersonal trust on prosocial tendencies in middle school students.

**Table 5:** Results of multiple regression analyses.

Variables	Negative legal emotion			Prosocial tendencies			Prosocial tendencies		
	*B*	SE	*t*	*B*	SE	*t*	*B*	SE	*t*
constant	71.35	4.209	16.95^***^	22.851	1.910	11.97^***^	59.993	5.145	11.66^***^
Interpersonal trust	0.268	0.054	5.003^***^	0.191	0.024	7.853^***^	0.173	0.058	2.968^**^
Negative legal emotion							0.497	0.136	3.667^**^
Gender	−0.568	1.696	−0.335	−2.005	0.769	−2.606^*^	0.428	1.678	0.255
Age	1.628	1.135	1.434	−0.116	0.515	−0.226	1.685	1.108	1.521
*R* ^2^	0.097			0.207			0.143		
*F*	9.018^***^			21.956^***^			10.460^***^		

* *p < 0.1;*

** *p < 0.01; and*

*** *p < 0.001.*

#### Validation of the Mediating Role of Negative Legal Emotion

**Figure 2:**
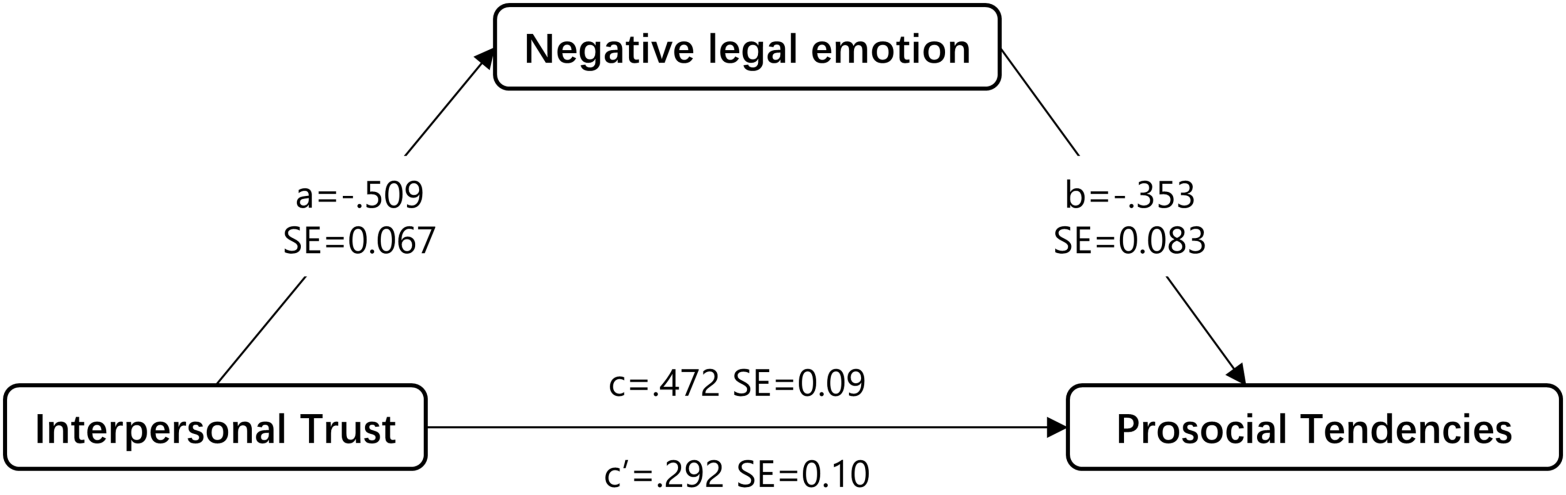
Hypothesized model with negative legal emotion as the mediator between interpersonal trust and prosocial tendencies for the entire sample. Unstandardized regression weights are presented. Controlled variables (age and gender) are omitted for clarity. All paths were significant at the *p* < 0.01 level.

Using PROCESS macro, we confirm that that self-esteem partially mediates the relation between interpersonal trust and prosocial tendencies (Figure 2): indirect effect = 0.180, SE = 0.053, 95% CI = [0.085, 0.293]. Therefore, the indirect effect is significantly different from zero at *p* < 0.05. The mediator could account for 38.1% of the effect.

## Discussion

The current study found that in our junior middle school sample, peer attachment predicts both positive and negative legal emotions. Moreover, we found that peer attachment affects legal emotion partially through self-esteem. The results also showed that negative legal emotion acted as a mediator in the relationship between interpersonal trust and prosocial tendencies. The model was gender and age-controlled, suggesting that the mediation worked in a similar way for both genders and across ages. The results provided important insights into the role of legal emotion, the emotional aspect of legal consciousness, in the social interactions and its potential psychological mechanism.

### The Legal Emotion

The problem of consciousness, its content, purpose, and functioning are key research fields of any social sciences, including the legal ones. Legal consciousness is a complex phenomenon that has various aspects of philosophical, legal, socio-political, and moral content. The structure of legal consciousness is a set of complex items. Cognitive, emotional, and conative components of legal consciousness are closely interrelated and perform certain functions: the cognitive component represents a set of legal knowledge; the emotional component is expressed in evaluative opinions on the law and its use; the conative component relates to the legal conceptions and orientations in behavior (Ratinov and Efremova, 1988). Due to its complexity, to date, however, there is no valid and reliable psychological assessment tool kit to give a holistic quantitative assessment of the level of legal consciousness. The difficulty in creating such an instrument is largely due to the insufficiently developed theoretical architecture of the legal consciousness, i.e., inability of researchers to agree upon legal consciousness’s universal and broadly accepted definition (Horák et al., 2021). Therefore, at present, all available instruments are aimed at the measurement of certain aspects of legal consciousness: its cognitive component, as well as the emotional and behavioral ones. The present study focused on the emotional aspect of legal consciousness.

Criminologists (e.g., Benson and Livelsberger, 2012), legal scholars (Bandes and Blumenthal, 2012), and psychology and law scholars (Wiener et al., 2006) have recently recognized the importance of emotion as a predictor of behavior. The legal emotion reflects the overall emotional assessment of law as a sociocultural phenomenon. The author’s previous study (Xu, 2020) has been focused on the differences along the valence dimension, i.e., negative, or positive attitudes toward the respondents’ assessment of law as a sociocultural phenomenon. A growing trend in legal consciousness research is to give them more weight in the development of legal consciousness than previously assumed and to view the role of one’s emotions with the culturally embedded sense of self (Abrego, 2011; Liu, 2017; Tungnirun, 2017). Given the strong relationship between emotion and the sense of self, the emotional aspect of legal consciousness, in comparison with the cognitive or knowledge aspect of legal consciousness, would be a better candidate for the investigation of the interaction of the legal consciousness and other social-cultural factors related to the self-consciousness. In the present study, we investigated (1) the potential factors which might affect legal emotion; and (2) the potential effect of legal emotion on other self-related social variables.

### Pathway to Legal Emotion in Early Adolescence: The Role of Self-Esteem and Peer Attachment

As mentioned in the introduction section, it is established that there is a strong relationship between security in attachment and self-esteem, both for peer attachment and parent attachment (Gorrese and Ruggieri, 2013), and there is a strong relationship between rational self-esteem and emotional response: either positive emotions such as empathy (Laible et al., 2004), or negative emotions such as depression (Ju and Lee, 2018) in adolescents. The findings of the current study indicated that self-esteem plays a partial mediating role in the process by which peer relationships influence legal emotion: good peer relationships, as a favorable condition of social support, can help individuals form good legal emotions, either positive or negative valenced. Self-esteem mediated this effect.

The theoretical background of the mediating role of self-esteem in the connection between peer relationships and other social behavior can be found in theories about the link between self-esteem and social relationships, which may be categorized into self-esteem antecedent or self-esteem consequence models (Marshall et al., 2014; Harris and Orth, 2020). Self-esteem sociometer theory proposes that personal experiences of social acceptance and rejection result in changes in the person’s state of self-esteem, whereas consequence models argue that social relationships predict the quality of self-esteem, which covers attachment theory. In this sense, peer attachment directly changes self-esteem, and the effects then propagate into other social behaviors, such as the legal emotion in our study.

We note that the mediation effect we found is partial instead of total. The partial mediation effect demonstrated that self-esteem in the peer-attachment→self-esteem→legal emotion route can only explain a fraction of the legal emotion phenomenal. Further investigation is definitely needed to arrive at a more comprehensive conclusion on it.

### The Mediating Role of Negative Legal Emotion in the Process by Which Interpersonal Trust Influence Prosocial Tendencies

We demonstrated that peer relationships can positively affect legal emotion and this effect is partially mediated by self-esteem. A following-up question is does legal emotion mediate certain effects between peer relationships and other social-cultural behavior? As described in the introduction section, in this preliminary investigation we selected interpersonal trust and prosocial tendencies as research targets.

Interpersonal trust is a measure of peer relationships, whereas prosocial tendencies are strongly affected by the interpersonal trust. The mediation effect of negative legal emotion found by the present study demonstrated that the legal emotion actively interacts with other social-cultural psychological structures. Therefore, it is one of the psychological modules which develops together with other important self-related internal psychological structures. The structure and the effect of legal emotion could be a potentially interesting topic for further studies.

### Implications

The development of legal consciousness reflects the legal socialization of the subjects. In this sense, understanding the factors affecting the development of legal consciousness and the factors affected by legal consciousness will greatly contribute to a better understanding of the legal socialization processes of adolescents. In the present study, we found that peer attachment would be a key factor affecting the emotional aspect of legal consciousness. For early adolescents who are in the developmental stage in which they are shifting from parent attachment to peer attachment. We further showed that self-esteem also plays an important role between these two variables. These results could have potential theoretical implications for a better understanding of legal socialization and general socialization, and might also have potential educational applications as we might be able to better construct the adolescent’s legal emotion *via* promoting peer attachment and self-esteem.

The finding that legal emotion mediates the effect of interpersonal trust on prosocial tendencies indicated that the legal emotion actively interacts with other self-related psychological structures. These interactions might have potential theoretical implications and could lead to a better understanding of the process of legal socialization.

### Limitations and Future Directions

We would like to acknowledge several methodological issues surrounding this study. First, although the sample size is large, one must be reminded that all the samples originate from the same city, same race, and same culture. Importantly, legal and moral concepts vary across cultures, and their development has different content and meanings in different cultures. In this sense, the present results are only generalizable to the eastern culture. It is well known that Eastern culture is more group-oriented, whereas Western culture is more individual-centered. This is consistent with the present finding that peer attachment predicts the emotional aspect of legal consciousness, nevertheless caution should be taken when generalized the results into Western culture. Follow-up studies can consider the selection of more diversified regional and cultural samples. Second, the present study was a preliminary examination of emotion in legal socialization using cross-sectional data. The cross-sectional nature of the present study limited the extension of our results. Future studies should adopt a longitudinal research design to collect data at multiple time points, which may enable a more in-depth examination of the relationship between variables. Third, the present study mainly focused on the general scores of the questionnaires, without examining the relationship between each sub-dimension of measurements. Finally, each variable is dependent upon students’ self-reporting. As such, there may be deviations or inaccuracies present. Combining these tests with behavior observation, parental reporting, school evaluations as well as other methods can help researchers obtain more objective and accurate information on the topic. Future studies should focus on these issues.

## Conclusion

In the present study, we found that for early adolescents, peer attachment predicts both positive and negative legal emotions. Moreover, we found that peer attachment affects legal emotion partially through self-esteem. Our results also showed that negative legal emotion acted as a mediator in the relationship between interpersonal trust and prosocial tendencies. The results provided important insights into the role of legal emotion, the emotional aspect of legal consciousness, in the social interactions and its potential psychological mechanism.

## Data Availability Statement

The raw data supporting the conclusions of this article will be made available by the authors, without undue reservation.

## Ethics Statement

The studies involving human participants were reviewed and approved by Wenzhou University Ethics Committee. Written informed consent for participation was not required for this study in accordance with the national legislation and the institutional requirements.

## Author Contributions

SX conceived the project. SX and LF carried out research and data analysis. LF and CS contributed in data analysis. SX and CS prepared the manuscript. All authors contributed to the article and approved the submitted version.

## Funding

This work was sponsored in part by the National Social Science Fund of China grant no. 21ZDA028 and Zhejiang Provincial Natural Science Foundation of China grant no. LQ19C090002 awarded to SX.

## Conflict of Interest

The authors declare that the research was conducted in the absence of any commercial or financial relationships that could be construed as a potential conflict of interest.

## Publisher’s Note

All claims expressed in this article are solely those of the authors and do not necessarily represent those of their affiliated organizations, or those of the publisher, the editors and the reviewers. Any product that may be evaluated in this article, or claim that may be made by its manufacturer, is not guaranteed or endorsed by the publisher.
